# Evaluation of a Pharmacist-Led Remote Warfarin Management Model Using a Smartphone Application (Yixing) in Improving Patients’ Knowledge and Outcomes of Anticoagulation Therapy

**DOI:** 10.3389/fphar.2021.677943

**Published:** 2021-07-01

**Authors:** Shudan Jiang, Qiuyi He, Jiajia Yan, Liyan Zhao, Yifan Zheng, Pan Chen, Xiao Chen

**Affiliations:** ^1^ Department of Pharmacy, The First Affiliated Hospital, Sun Yat-Sen University, Guangzhou, China; ^2^ Department of Pharmacy, The Maternal and Child Health Care Hospital of HuaDu District (Huzhong Hospital), Guangzhou, China

**Keywords:** remote management, warfarin, smartphone application, pharmacist, anticoagulation therapy

## Abstract

**Background:** The management of warfarin-treated patients has been recognized as a challenge due to narrow therapeutic range and food and drug interactions in warfarin therapy. We aim to evaluate the effect of a pharmacist-led remote warfarin management model using a smartphone application (app) on anticoagulation therapy.

**Methods:** Eligible patients who had received warfarin therapy after mechanical heart valve replacement were enrolled. The intervention group was offered a pharmacist-led remote warfarin management model using the app named Yixing. Yixing incorporates functions including automatic daily reminder, personal health record, educational program, and online counseling. The control group received traditional pharmacy services without Yixing. Co-primary outcomes were patients’ awareness score of warfarin therapy obtained from questionnaire, the medication adherence measured by the percentage of the correct-warfarin-taken days in the monitored period, the fraction of time in therapeutic range (FTTR), and the incidence of anticoagulation-related complications. The needed information of the patients was acquired *via* electronic medical records from the hospital, Yixing system and telephone follow-up when necessary.

**Results:** 64 and 66 patients were initially in the intervention and control groups respectively. After propensity score matching, 50 patients were assigned in each group. The intervention group had a median age of 51.0 years, in which 27 (54%) were male. The control group had a median age of 50.5 years, in which 28 (56%) were male. Patient awareness score in the intervention group was 8.00 (2.00), which was higher than that in the control group, with score at 6.50 (2.50) (*p* = 0.001). No significant difference was found in the percentage of the correct-warfarin-taken days between the two groups (*p* = 0.520). The median (interquartile range) value of FTTR was 80.3% (21.9%) and 72.1% (17.7%) in the intervention and control groups respectively (*p* = 0.033), and no significant differences in the incidence of anticoagulation-related complications were observed (*p* = 0.514).

**Conclusion:** The pharmacist-led remote warfarin management model using Yixing improves patients’ awareness of warfarin therapy and increases FTTR, but may not have significant improvements on medication adherence and safety.

## Introduction

Anticoagulation therapy plays a crucial role in the prevention and treatment of diseases such as venous thromboembolism, atrial fibrillation, and valvular heart disease. Although new anticoagulants such as rivaroxaban and dabigatran have been shown with increasing utility, the vitamin K antagonist warfarin remains the mainstream in treating thromboembolic diseases, especially for the patients undergoing mechanical heart valve replacement (Eikelboom et al., 2013). In China, there are over 200,000 patients accepting heart valve replacement operation each year, generating a large requirement of warfarin use (Luo, 2010). However, the warfarin use in clinical practice is still a challenge due to its narrow therapeutic range and complex food and drug interactions. Thus, warfarin needs to be prescribed individually and monitored according to international normalized ratio (INR).

Inpatients receive routine INR monitoring and subsequent dose adjustment of warfarin under the supervision of physicians, nurses, and pharmacists to ensure the effectiveness and safety of warfarin. For discharged patients, effective follow-up anticoagulation management is critical for controlling INR in a steady state to lower the risk of drug-related adverse events and improve medication adherence. Remote follow-up management of anticoagulation therapy has been developed in the past decades (Waterman et al., 2001; Bussey et al., 2013). In comparison to the remote management using telephone follow-ups, public social platforms and remote electronic service systems, management using smartphone application (app) has drawn great interest in recent years, due to their functions in timely recording warfarin dose and INR value, and return of individualized medication recommendation (Winkle et al., 2014). However, most of those apps were operated commercially without enough participation of medical staffs, and the reported medical staffs responsible for follow-up management were normally physicians and nurses, focusing on the controls of diseases progress and medication adherence. Till now, little has been reported about the role of pharmacists in the remote management. The management team enrolling pharmacists would improve the rational use of warfarin and lower the risk of drug-related adverse events (Saokaew et al., 2010; Marcatto et al., 2018). In the present study, the app (named as Yixing) was developed and managed by hospital pharmacists specialized in the anticoagulation therapy. The purpose of this study was to comprehensively evaluate the effects of pharmacist-led remote management model on patients’ awareness on warfarin therapy, medication adherence, anticoagulation efficacy and safety.

## Methods

### Study Design

This was a prospective study enrolling patients who had mechanical heart valve replacement in the department of cardiac surgery, the First Affiliated Hospital of Sun Yat-Sen University, and discharged between January 2017 and June 2019. The study was approved by the Ethic Committee for Clinical Research and Animal Trails of the First Affiliated Hospital of Sun Yat-Sen University.

### Inclusion and Exclusion Criteria

The inclusion criteria were: 1) patients undergoing mechanical heart valve replacement; 2) firstly initiating warfarin treatment. The exclusion criteria were: 1) patients who had any serious chronic diseases, including cerebral infarction or cerebral hemorrhage, coronary heart disease, and severe lung, liver, or renal dysfunction; 2) follow-up period less than six months, due to the reasons such as unwilling to provide needed information anymore or died of diseases unrelated to anticoagulation.

### Intervention

In the intervention group, each patient was instructed to install and use the app, then offered continuous medication education by hospital pharmacists specialized in anticoagulation drug therapy via the app during hospitalization and at least four times after discharge. In contrast, the patients in the control group did not install the app, and received routine oral medication education by pharmacists during hospitalization, but did not get any support after discharge, and the needed information during follow-up period were obtained *via* telephone survey. Besides the function in electronic medication education, Yixing incorporates functions including automatic daily medication reminder, personal health record and online counseling. When patients confirmed taking that day’s dose, a time-stamped event was recorded. Patients were instructed by pharmacists to input their medical history, data of anticoagulation drugs and laboratory tests (INR was compulsively demanded), and the pharmacists checked patients’ health records regularly (Supplementary Figure S1). Moreover, patients were free to contact pharmacists in forms of text, audio or pictures any time when having questions related to anticoagulation therapy (Supplementary Figure S2).

### Follow-Up Assessment

The follow-up assessment period was 6 months. Co-primary outcomes were patient awareness score, medication adherence, the fraction of time in therapeutic range (FTTR), and the incidence of anticoagulation-related complications. Data in the intervention group were obtained from the hospital electronic medical system and Yixing system, and data in the control group were obtained via hospital electronic medical system and telephone follow-up. The knowledge questionnaires were administered at the end of six-month-long follow-up period. To ensure the quality of telephone surveys, patients themselves were required to answer the phone if there was no language barrier, and the questions were ordered in a logical and patient-friendly sequence. Open questions were adopted in the questionnaire of patients’ knowledge on warfarin therapy to avoid the influence of hint effect of the options (Supplementary Table S1).

Medication adherence was defined as taking warfarin according to medical advice (frequency, dosage, and precautions) and was calculated by the percentage of days that the correct warfarin dose was taken in the monitored period. Patients who forgot to take the medicine at the set time but remember to take later in that day was also regarded as a correct dose. For the intervention group, the warfarin dose records were obtained through self-report in the app, and were confirmed via telephone each month; for the control group, the warfarin dose records were all obtained *via* telephone survey each month. The patients in the intervention group needed to record INR for at least three times on the app after discharge, and the patients in the control group needed to provide at least 3 INR test results *via* the telephone survey. INR target range was set at 1.8∼2.5 for warfarin monitoring. FTTR was calculated using linear interpolation as described by Rosendaal et al. (1993).

### Statistical Analysis

The sample size was calculated according to the expected difference of the three quantitative outcomes (patients’ awareness, medication adherence, and anticoagulation efficacy) between the traditional and new anticoagulation management model groups. Based on our preliminary research, the difference was estimated to be three quarters of standard deviation. A total of 74 patients will be needed based on a power of 90% and a two-tailed z-test with α = 0.05. Considering a 20% loss to follow-up, 93 patients will be required. Finally, we decided that at least 100 patients should be enrolled in the study to provide the power required to identify the expected difference under the assumptions. Due to a wide variation in the prevalence of reported clotting and bleeding episodes, we did not perform power calculations of anticoagulation safety.

A propensity score-matched analysis was used to minimize the baseline bias between the two groups, according to a guideline of propensity score matching (Yao et al., 2017). The propensity score was derived from a multivariable logistic model including all the variables: age, sex, race, marital relation, education, employment, residence area, whether living along, surgery, insurance, affordability, level of hospital, travel time, and whether using a drug reminder. According to the propensity score, the intervention patients were matched 1:1 without replacement to the control patients by using nearest-neighbor matching within a caliper set at 0.05.

Continuous data were described as mean ± standard deviation (SD), and skewed data were expressed as median (interquartile range). Categorical variables were described as number and proportion. The comparison of patients’ baseline, knowledge, adherence, therapy efficacy, and safety were performed using the Wilcoxon rank sum test, the Chi square test or Fisher’s exact test (as appropriate). A 2-sided *p* value < 0.05 was considered statistically significant. All statistical analyses were performed using the Statistical Product and Service Solutions version 25.0 (IBM Corp., United States).

## Results

### Patient Characteristics

64 patients and 66 patients were initially involved in the intervention group and the control group, respectively. After PSM, 50 patients were in the intervention group and had a median age of 51.0 years, in which 27 (54%) were male and a corresponding 50 patients were in the control group and the median age was 50.5 years, in which 28 (56%) were male. The characteristics of the patients before and after the propensity score matching were shown in Table 1, and there were no statistically significant differences between the two groups.

**TABLE 1:** Baseline Characteristics of enrolled patients before and after propensity-score matching.

	Pre-matching			Post-matching		
	Intervention (*n* = 64)	Control (*n* = 66)	*p* value	Intervention (*n* = 50)	Control (*n* = 50)	*p* value
Age, y, median (interquartile range)	51.0 (17.0)	49.5 (18.0)	0.341	51.0 (16.0)	50.5 (18.0)	0.855
Male, *n* (%)	38 (59%)	34 (52%)	0.469	27 (54%)	28 (56%)	1.000
Han, *n* (%)	64 (100%)	65 (99%)	1.000	50 (100%)	50 (100%)	—
Married, *n* (%)	60 (94%)	61 (92%)	0.554	48 (96%)	47 (94%)	1.000
Education, *n* (%)	—	—	0.635	—	—	0.738
No formal education	6 (9%)	6 (9%)	—	3 (6%)	5 (10%)	—
Primary school	15 (23%)	9 (14%)	—	12 (24%)	7 (14%)	—
Junior high school	22 (34%)	23 (35%)	—	19 (38%)	20 (40%)	—
Senior high school	9 (14%)	13 (20%)	—	7 (14%)	9 (18%)	—
Higher education	12 (19%)	15 (23%)	—	9 (18%)	9 (18%)	—
Employment, *n* (%)	30 (47%)	31 (47%)	1.000	21 (42%)	23 (46%)	0.840
Residence, *n* (%)	—	—	0.759	—	—	0.759
Urban	43 (67%)	47 (71%)	—	33 (66%)	34 (68%)	—
Countryside	21 (33%)	19 (29%)	—	17 (34%)	16 (32%)	—
Living alone, *n* (%)	2 (3%)	4 (6%)	0.680	2 (4%)	2 (4%)	1.000
Surgery, *n* (%)	—	—	0.840	—	—	0.260
AVR	19 (30%)	20 (30%)	—	14 (28%)	17 (34%)	—
MVR	27 (42%)	28 (42%)	—	25 (50%)	19 (38%)	—
DVR	15 (23%)	17 (26%)	—	9 (18%)	14 (28%)	—
Bentall’s	3 (5%)	1 (2%)	—	2 (4%)	0 (0%)	—
Insurance, *n* (%)	—	—	0.129	—	—	0.080
Commercial	1 (2%)	0 (0%)	—	1 (2%)	0 (0%)	—
Basic urban workers	31 (48%)	37 (56%)	—	26 (52%)	23 (46%)	—
Basic urban residents	6 (9%)	2 (3%)	—	6 (12%)	2 (4%)	—
New rural cooperative	23 (36%)	27 (41%)	—	15 (30%)	25 (50%)	—
None	3 (5%)	0 (0%)	—	2 (4%)	0 (0%)	—
Affordability, *n* (%)	63 (98%)	62 (94%)	0.366	49 (98%)	47 (94%)	0.617
Level of hospital, *n* (%)	—	—	0.843	—	—	0.867
Primary	9 (14%)	7 (11%)	—	6 (12%)	4 (8%)	—
Secondary	16 (25%)	17 (26%)	—	13 (26%)	13 (26%)	—
Tertiary	39 (61%)	42 (64%)	—	31 (62%)	33 (66%)	—
Travel time, *n* (%)	—	—	0.725	—	—	0.449
＜30 min	46 (72%)	50 (76%)	—	37 (74%)	35 (70%)	—
30∼60 min	10 (16%)	10 (15%)	—	8 (16%)	9 (18%)	—
60∼120 min	6 (9%)	6 (9%)	—	3 (6%)	6 (12%)	—
＞120 min	2 (3%)	0 (0%)	—	2 (4%)	0 (0%)	—
Using a drug reminder, *n* (%)	41 (64%)	46 (70%)	0.577	35 (70%)	36 (72%)	1.000

AVR: aortic valve replacement; MVR: mitral valve replacement; DVR: double valve replacement; Affordability: patients can afford the cost of anticoagulation therapy by themselves; Level of hospital: the medical institutions where patients have INR tests after discharge; Travel time: time spending on travel to the hospital.

### Patients Awareness on Warfarin Therapy

The median (interquartile range) score of the patient awareness in the intervention group was 8.00 (2.00), which was significantly higher than that in the control group, with a score at 6.50 (2.50) (*p* = 0.001). As shown in Table 2, there were more patients who scored higher than seven points in the intervention group than in the control group (66 vs. 38%, respectively), and the percentage of the patients with a score higher than 8.5 points in the intervention group was three times higher than that of the control group.

**TABLE 2:** Score distribution of patient awareness on warfarin therapy.

Score	Intervention	Control
(1, 2.5]	0 (0%)	1 (2%)
(2.5, 4]	1 (2%)	5 (10%)
(4, 5.5]	5 (10%)	10 (20%)
(5.5, 7]	11 (22%)	15 (30%)
(7, 8.5]	18 (36%)	14 (28%)
(8.5, 10]	15 (30%)	5 (10%)
	50 (100%)	50 (100%)

### Medication Adherence

No significant difference was found in the percentage of the correct-warfarin-taken days between the two groups (*p* = 0.520). 72% of patients in the intervention group and 78% of patients in the control group had 100% correct-warfarin-taken days (Table 3).

**TABLE 3:** Patients’ distribution in the percentage of the correct-warfarin-taken days.

Percentage	Intervention	Control
(96%, 97%]	0 (0%)	1 (2%)
(97%, 98%]	1 (2%)	2 (4%)
(98%, 99%]	3 (6%)	0 (0%)
(99%, 100%)	10 (20%)	8 (16%)
100%	36 (72%)	39 (78%)
	50 (100%)	50 (100%)

### Anticoagulation Efficacy and Safety

FTTR was 80.3% (21.9%) and 72.1% (17.7%) in the intervention and the control groups, respectively (*p* = 0.033). Similar numbers of patients in the two groups achieved the range of 70–90%, while the percentage of the intervention patients in the 90–100% FTTR range was more than twice as that in the control patients (Table 4).

**TABLE 4:** Patients’ distribution in the FTTR.

FTTR	Intervention	Control
(10, 30%]	1 (2%)	1 (2%)
(30%, 50%]	4 (8%)	6 (12%)
(50%, 70%]	11 (22%)	16 (32%)
(70%, 90%]	25 (50%)	23 (46%)
(90%, 100%]	9 (18%)	4 (8%)
	50 (100%)	50 (100%)

No serious hemorrhage or thromboembolism events occurred in all of the patients during the follow-up period, and there were no significant differences in the incidences of non-serious bleeding and embolism between the two groups (*p* = 0.514) (Table 5).

**TABLE 5:** Anticoagulation-related complications.

	Non-serious bleeding and embolism			Serious hemorrhage or thromboembolism events	Dizziness	Total
	Gingival bleeding	Skin ecchymosis	Gastrointestinal and urinary bleeding			
Intervention	3 (6%)	1 (2%)	0 (0%)	0 (0%)	1 (2%)	5 (10%)
Control	6 (12%)	2 (4%)	2 (4%)	0 (0%)	1 (2%)	11 (22%)

Dizziness: a sensation of whirling or falling.

### Subgroup Analysis

Subgroup analyses were carried out basing on age, sex, educational level and patients’ region of residence (Table 6). The effect of the model on medication adherence was similar among specific subsets of the population. The model significantly increased patients’ awareness regardless of sex and region, but there were distinctions in different age and educational level groups. Patients above 50 years old in the intervention group scored two points higher than their counterparts (*p* = 0.001) while the gap was not obvious in patients below 50 years old (*p* = 0.109). Patients who had accepted at least primary school or junior high school level of education performed better in the questionnaire under the management of remote model (*p* = 0.001) while patients in other educational level groups did not show significant advantages over the control group.

**TABLE 6:** Subgroup analyses of the differences between two groups.

	Knowledge			Adherence			FTTR		
	Intervention	Control	*p*	Intervention	Control	*p*	Intervention	Control	*p*
Age	—	—	—	—	—	—	—	—	—
below 50	8.50 (2.00)	7.50 (2.00)	0.109	1.00 (0.00)	1.00 (0.00)	0.765	0.84 (0.19)	0.73 (0.16)	0.026
At or above 50	8.00 (2.38)	6.00 (2.25)	0.001	1.00 (0.00)	1.00 (0.00)	0.201	0.77 (0.28)	0.71 (0.27)	0.307
Sex	—	—	—	—	—	—	—	—	—
Female	7.50 (2.00)	6.50 (2.00)	0.015	1.00 (0.01)	1.00 (0.00)	0.320	0.75 (0.17)	0.70 (0.20)	0.037
Male	8.50 (2.50)	7.00 (3.00)	0.010	1.00 (0.00)	1.00 (0.00)	0.965	0.85 (0.28)	0.75 (0.20)	0.170
Education	—	—	—	—	—	—	—	—	—
No formal education	6.00 (0.00)	6.50 (3.50)	0.647	1.00 (0.00)	1.00 (0.00)	0.845	0.50 (0.00)	0.68 (0.18)	0.101
≥primary school	8.00 (2.00)	7.00 (2.50)	0.001	1.00 (0.00)	1.00 (0.00)	0.576	0.81 (0.21)	0.72 (0.20)	0.016
≥Junior high school	8.50 (2.00)	7.00 (2.50)	0.001	1.00 (0.00)	1.00 (0.00)	0.380	0.81 (0.21)	0.72 (0.18)	0.108
≥Senior high school	8.75 (1.88)	7.75 (3.13)	0.193	1.00 (0.01)	1.00 (0.00)	0.284	0.88 (0.18)	0.79 (0.21)	0.125
≥Higher education	9.00 (2.00)	7.50 (3.50)	0.722	1.00 (0.01)	1.00 (0.00)	0.696	0.88 (0.19)	0.75 (0.22)	0.270
Residence	—	—	—	—	—	—	—	—	—
Urban	8.50 (2.00)	7.00 (2.13)	0.008	1.00 (0.00)	1.00 (0.00)	0.987	0.84 (0.21)	0.72 (0.20)	0.106
Countryside	8.00 (2.75)	5.75 (2.38)	0.026	1.00 (0.01)	1.00 (0.00)	0.268	0.80 (0.30)	0.70 (0.25)	0.097

As for the effect on anticoagulation efficacy, the model functioned better in the younger age group (*p* = 0.026) and the female (*p* = 0.037), and there was a statistically significant difference among patients who had accepted at least primary school level of education (*p* = 0.016).

## Discussion

Our study demonstrated that the Yixing app improved patient awareness of warfarin therapy, which was mainly attributed to the increased number of patients with a high score (higher than seven points). Further subgroup analysis proved that the app was especially helpful among the elderly, which may be explained by that the elder population in China was less likely to search for the anticoagulation-related knowledge on their own initiative since a large proportion of them were not familiar with the modern internet and search engine. Education was also a significant factor influencing patients’ awareness score. Patients with a fundamental educational background may better understand the anticoagulation, and then memorize the warfarin knowledge offered in the model. This positive correlation between education and the score was also revealed by other studies. In an Iranian study, 150 patients were evaluated *via* an Anticoagulation Knowledge Assessment (AKA) questionnaire consisting of 29 questions, from which researchers found the level of general education and socioeconomic factors were important elements determining the patient awareness (Pourafkari et al., 2018). However, there was also a study proposing that education was a non-significant factor for knowledge improvement (Shilbayeh et al., 2019). The discrepancy may result from the different design of the knowledge assessment questionnaires and enrolled patients from different countries.

It is considered that patients having higher anticoagulation therapy knowledge are more likely to recognize the importance of the treatment and then maintain better medication adherence (Wang et al., 2014). However, although Yixing users had a better understanding of warfarin therapy, patients in both groups had high percentage of the correct-warfarin-taken days, and there was no significant difference between them. Differing from drugs for the treatment of other chronic diseases such as hypertension and diabetes, warfarin withdrawal may lead to devastating consequences, so the patients tend to take warfarin on time. In the telephone surveys, few patients refused to take warfarin, and the missed doses always happened unintentionally. According to those who had forgotten taking warfarin, their missed doses always occurred in the early stage of treatment when the habit of taking medicine on time had not been established stably, and after they found appropriate solutions, like preparing warfarin in the workplace, their medication adherence improved over time. Moreover, the no significant difference in the medication adherence may be related with the low period of follow-up assessment. We evaluated patients’ medication adherence at the time point of 6 months, which may not be long enough to show the impact of Yixing because patients without continuous remote management may not keep on such great medication adherence throughout the lifelong period of the treatment, especially when they feel like their condition is under control years later.

FTTR is a common-used primary outcome variable for assessing the effect of anticoagulation management (Samsa and Matchar, 2000), though its reliability could sometimes be influenced by factors like access to care, payment for care and recheck frequency (Reiffel, 2017). Patients who have easier access to care and more sufficient medical budget are more likely to have their INR test at a recommended recheck frequency, which may lead to a more accurate calculation result of FTTR. Our data demonstrated that there were no statistically significant differences in these factors between the two group (Travel time *p* = 0.449, Affordability *p* = 0.617). According to the previous reports using remote warfarin management tools, the FTTR ranged from 40 ± 21%–75 ± 22% (Miyamoto et al., 2015; Li et al., 2019). In our study, the patients using Yixing got a median FTTR of 80.3%, which was attributed by the increase in the number of high-proportion patients (90–100%). Improved patient knowledge was reported resulting in better anticoagulation control (Nasser et al., 2011; Wang et al., 2014; Cao et al., 2020), but the conclusion seemed not applicable in our study. The younger patients under the model did not show a big advantage in the aspect of anticoagulation knowledge but maintained higher FTTR while the elderly showed the opposite. The discrepancy also occurred when looking at other categories. It may result from the inconsistency between patients’ expressed awareness and their actual behavior, the limited number of patients in certain subsets, and the inherent defect of the current main calculation method of the FTTR. Furthermore, the app users and the control patients had similar medication adherence but showed different anticoagulation efficacy. The relationship between medication adherence and good anticoagulation level was also uncertain. Some found that adequate adherence was significantly associated with anticoagulation control (Davis et al., 2005; Kimmel et al., 2007; Wang et al., 2014), while others reported medication adherence did not predict therapeutic anticoagulation control (Kim et al., 2011; Mayet. 2016). This might because in addition to adherence, the response to warfarin can be affected by other factors like interference of concomitant drugs, alcohol intake, and vitamin K in the diet (Kim et al., 2011).

In our study, no significant difference was found in the incidence of anticoagulation-related complications between the two groups, which was largely consistent with a meta-analysis, which found that there were no significant differences in the incidence of major or minor bleeding events, thromboembolic events, and warfarin-related emergency department visits between online and hospital management (Xia et al., 2018).

### Strengths

Compared with normal remote managements like telephone follow-up, the Yixing app established a communication platform for both patients and pharmacists, facilitating in-time warfarin management and enhancing the efficiency. Although there were also studies reporting warfarin management apps (Miyamoto et al., 2015; Smaradottir et al., 2018; Li et al., 2019; Shilbayeh et al., 2019), the Yixing app incorporates more functions, including medication education, automatic daily medication reminders, personal health records and online counseling. Besides, the effect of the pharmacist-led remote management model using Yixing in the anticoagulation therapy was more comprehensively evaluated, not only focusing on FTTR, but also evaluating patient awareness of warfarin therapy and medication adherence.

### Limitations

There are several limitations of this study. Firstly, consecutive follow-up assessments at time points like 6, 12, 18, 24, and 30 months after enrollment should have been performed to better identify the changes in the co-primary outcomes (especially the safety outcome) and a larger sample size was needed for a more accurate subgroup analysis result. Secondly, the participants were not randomly assigned to the two groups in this study, although we tried to balance the baseline bias using propensity score matching, randomized clinical trial needs to be performed further to confirm the result. Finally, adherence was not assessed in same manner between the two groups, which may be an inherent shortcoming of this kind of remote management model study. Although some strategies were performed to minimize the influence, for example, the data collections via telephone in the control group and via app in the intervention group were made at the same time, this issue still needs to be noticed in the further study.

## Conclusions

The pharmacist-led remote warfarin management model using the Yixing app improves patient awareness of warfarin therapy and increases FTTR, but may not have significant improvements on medication adherence and safety.

## Data Availability

The raw data supporting the conclusions of this article will be made available by the authors, without undue reservation.
