# Supplementary material for: Evaluation of a Pharmacist-Led Remote Warfarin Management Model Using a Smartphone Application (Yixing) in Improving Patients’ Knowledge and Outcomes of Anticoagulation Therapy
Source: Front Pharmacol. 2021 Jul 1;12:677943. doi: 10.3389/fphar.2021.677943 (PMC8281133; doi:10.3389/fphar.2021.677943)
Supplement: Supplementary file 1 [file DataSheet1.docx]

Supplementary Material


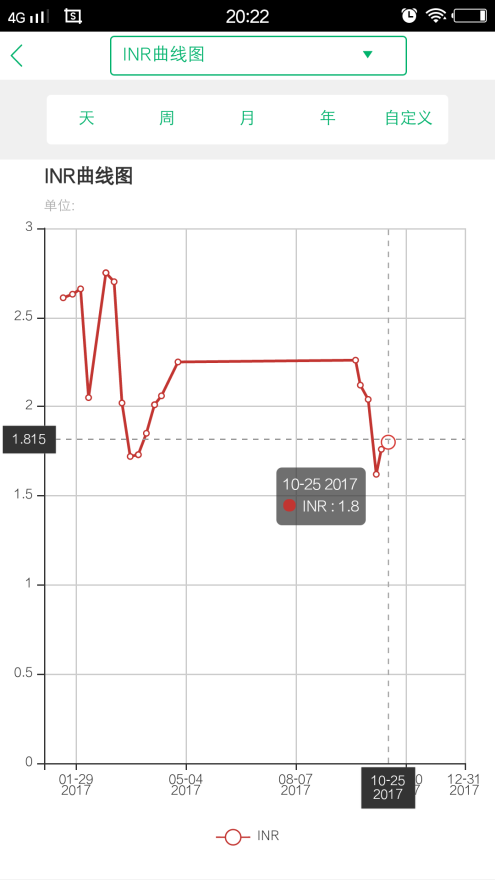

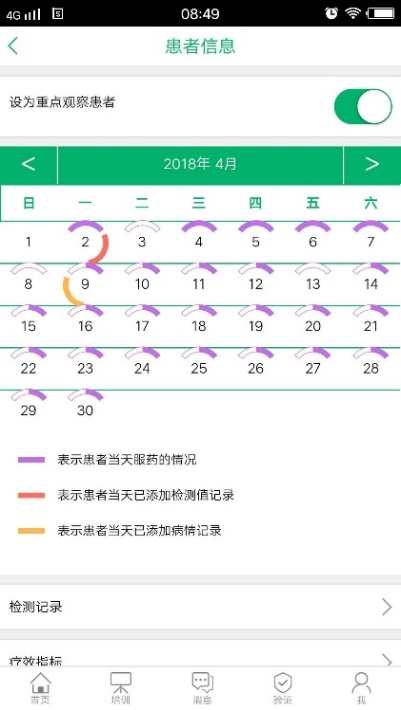


**A B**

**Supplementary Figure 1.** Health records

A. INR line graph from one of the patients in the study. the showing period is user-defined.

B. Pharmacists may check the health records of patients. There will be a mauve indicator if the patient confirms taking that day’s dose, and the length of curve will turn half if the patient adds another medication plan. For example, besides warfarin, the patient also needs to take atorvastatin. There will be a red indicator if the patient records a laboratory test result. There will be a yellow one if the patient records a disease-related condition, such as bleeding or embolism event.


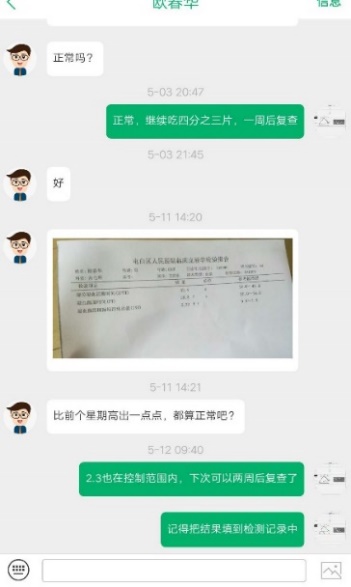


2.3 is within the target range and the next check can be arranged two weeks later. Please remember to record this result.

INR now is a little higher than that in last week, is this result normal?

Yes. Continue to take three quarters of the tablet and check next week.

OK.

Is the result normal?

**Supplementary Figure 2.** Online consulting

**Supplementary Table 1.** Questionnaire about patients’ knowledge on warfarin therapy

1. What is the effect of warfarin?

1 point for “anticoagulation or thromboprophylaxis”, otherwise 0 point.

2. What is the common side effect of warfarin?

1 point for “bleeding by overmedication (or thrombus from insufficient anticoagulant therapy)”, otherwise 0 point.

3. When the dose of warfarin is stable, how often do you think the laboratory test of coagulation parameters are required?

1 point for “every one or two months, or according to the previous INR results”, otherwise 0 point.

4. Which item do you need to pay attention to in coagulation parameters?

1 point for “INR (and PT)”, otherwise 0 point.

5. Do you know your INR target range?

1 point for “1.8~2.5”, otherwise 0 point.

6. During warfarin treatment, what should be paid attention to in terms of diet?

1 point for accurate and comprehensive answers, such as “green vegetables, broccoli, spinach, grapefruit and animal offal”. 0.5 point for not comprehensive but accurate answers, like only listing one or two food. 0 point for wrong answers.

7. Which vitamin can affect warfarin?

1 point for “vitamin K”, otherwise 0 point.

8. Do you think alcohol use will affect warfarin, and do you drink?

1 point for “Yes, it will” “No, I do not”, otherwise 0 point.

9. During warfarin treatment, what kind of bleeding do you think should be contacted with a doctor as soon as possible?

1 point for “gastrointestinal and urinary bleeding”. 0.5 point for including other slight type of bleeding. 0 point for wrong answers.

10. If you forget to take warfarin tonight, will you need to take it as soon as you remember tomorrow morning?

1 point for “No, just take the normal dose that night”, otherwise 0 point.

Note: The full score of the questionnaire was 10 points. Points will be given when patients express the correct meaning. Specific words do not need to be the same.
